# A fast, non-invasive, quantitative staining protocol provides insights in *Plasmodium falciparum* gamete egress and in the role of osmiophilic bodies

**DOI:** 10.1186/1475-2875-13-389

**Published:** 2014-10-01

**Authors:** Pablo Suaréz-Cortés, Francesco Silvestrini, Pietro Alano

**Affiliations:** Dipartimento di Malattie Infettive, Parassitarie ed Immunomediate, Istituto Superiore di Sanità, Viale Regina Elena n.299, 00161 Roma, Italy

**Keywords:** *Plasmodium falciparum*, Gametocytes, Gametogenesis, Parasite egress, Wheat germ agglutinin, Osmiophilic bodies, Mosquito transmission

## Abstract

**Background:**

Ability of *Plasmodium falciparum* gametocytes to become extracellular during gametogenesis in the mosquito midgut is a key step of the parasite life cycle. Reliable and quantitative measurement of the efficiency of gamete egress is currently constrained by the fact that this phenomenon is usually observed and quantified *in vitro* either by live microscopy, by statistically limited ultrastructural analysis or by surface antibody-based protocols which can interfere with this fast and complex cellular process.

**Methods:**

A protocol was developed based on fluorescent wheat germ agglutinin (WGA) surface staining of erythrocytes containing mature *P. falciparum* gametocytes. After a single centrifugation step and within minutes from the induction of gametogenesis, the activated gametes can be inspected for presence or absence of the fluorescent WGA staining of the host erythrocyte membrane and scored respectively as intracellular or emerged from the erythrocyte.

**Results:**

Gametogenesis and gamete egress from WGA surface stained, infected erythrocytes occur with normal kinetics and efficiencies. Quantitative measurements of gamete egress can be obtained in live and in paraformaldehyde-fixed cells, which validates this protocol as a suitable tool both for live imaging studies and for higher throughput applications. The protocol was used here to provide functional information on the ability of gametes to egress through a single exit point induced in the host red blood cell membrane, and to re-analyse the phenotype of Pfg377- and osmiophilic body-defective gametes, suggesting that such parasite components are not directly involved in disruption and shedding of the erythrocyte membrane in female gamete egress.

**Conclusions:**

The development of a reliable, fast, non-invasive and quantitative protocol to finely describe and to measure efficiency of *P. falciparum* gamete egress is a significant improvement in the tools for functional studies on this key process of the parasite life cycle. This protocol can be used to investigate the molecular mechanisms underlying gamete egress and its adaptation to high throughput applications will enable identification of transmission blocking inhibitors.

## Background

The observation that inhibition of *Plasmodium falciparum* transmission has beneficial effects on incidence and morbidity of malaria [1] boosted a renewed interest in the biology of parasite sexual stages and in the design of *Plasmodium* transmission blocking strategies. Improvement of available methodologies to dissect and quantitatively monitor cellular mechanisms of parasite sexual differentiation becomes an essential activity to identify critical parasite targets and to assess the efficacy of anti-transmission interventions.

The transformation of the intracellular *P. falciparum* mature gametocytes into extracellular gametes is definitely one of the most critical points and a significant bottleneck in the parasite life cycle [2–4]. In this process, which occurs within minutes from the uptake of the blood meal in the mosquito gut and that can be readily reproduced *in vitro*, elongated mature stage V gametocytes of both sexes initially become round shaped within the infected erythrocyte (‘rounding up’), then progressively disrupt the surrounding parasitophorous vacuole membrane (PVM) and the erythrocyte membrane to finally egress. During this process the male gametocyte undergoes a dramatic transformation leading to the production of eight haploid motile microgametes, whilst the female gamete retains its spherical shape after egress and can be fertilized by a male gamete to produce a zygote, which further transforms into an ookinete.

The molecular and cellular mechanisms responsible for gamete egress are comparatively less understood than those governing egress of merozoites at the burst of asexual schizonts. In *P. falciparum* treatment with protease inhibitors and ablation of specific parasite genes have been reported to block or inhibit this process in female gametes [5] and in gametes of both sexes [6, 7]. In *Plasmodium berghei* the process was blocked in male gametes defective for PbActinII or a perforin-like protein [8, 9] and in gametes of both sexes upon disruption of the *pbpeg3mdv1* or the *pbgest* genes [10, 11]. Analysis of gamete egress in several studies however largely relied on optical microscopy of live or fixed parasites or on examination of a limited number of independent ultrastructural sections of induced gametes. A reliable quantitative assessment of egress is not straightforward, particularly in the case of the spherical female gametes, and so far relied either on analysis of Giemsa-stained smears or on using two protocols based on cell surface staining with specific antibodies. In one protocol, fixed and reacted spherical gametes are inspected for presence or absence of surface reactivity to antibodies against the red blood cell surface molecule Band3 and respectively scored as intracellular or egressed [6, 12]. In the other method, fixed non-permeabilized gametes are analysed for surface reactivity to an antibody against the gametocyte/gamete surface antigen Pfs230, in this case with the positive cells scored as successfully egressed [5].

As both protocols require parasite fixation followed by incubations with primary and secondary antibodies and several washing steps, one concern was that such lengthy manipulations might alter integrity of the parasite and/or host cell membrane compartments, which are rapidly rearranged in gametogenesis, thus confounding the evaluation of egress. For this reason, a much faster and simpler protocol was developed to measure gamete egress, based on the labelling of infected erythrocytes prior to induction of gametogenesis. The protocol described here minimizes cell manipulation and experimental time prior to analysis and it is particularly suited to measure egress in female gametes, the most difficult to be unambiguously scored by optical microscopy. The new protocol was used here to closely follow disruption of the erythrocyte membrane in gamete egress and to further analyse the role in this process of osmiophilic bodies (OBs), the gametocyte secretory organelles abundant in female gametocytes.

## Methods

### *Plasmodium falciparum*parasites and cultures

Parasites from clones 3D7 and *pfg377KO* B7, E4 and G5 [5] were cultured in 0^+^ human red blood cells at 5% haematocrit in RPMI 1640 plus hypoxanthine 50 μg/ml, HEPES 25 mM, 0.225% sodium bicarbonate and 10 mg/ml gentamicin, supplemented with 10% heat inactivated human serum. Parasites were kept at 37°C, in a 2% O_2_, 5% CO_2_ and 93% N_2_ atmosphere. For gametocyte production, asynchronous parasites were grown to high parasitaemia (>8%) and culture medium was doubled at this point. The day after, medium was changed and N-acetylglucosamine 50 mM added. N-acetylglucosamine was maintained for three days until no asexual parasites were detected in the culture. Stage II gametocytes were detected 48 hours after the addition of N-acetylglucosamine while mature stage V appeared nine days after the start of the treatment.

### Erythrocyte surface labelling and gamete egress measurement

Cultures of red blood cells (RBC) uninfected or infected with mature stage V gametocytes were incubated with 5 μg/ml WGA-Alexa Fluor conjugate (Life Technologies) at 37°C for 15 min. Gametogenesis was then induced by pelleting the samples at 1,000 g for 1 min and rapidly resuspending cells in the selected conditions. For xanthurenic acid (XA) induction, 20 μM of the compound was added to complete medium with serum. In the case of gametogenesis induced by an increase in pH, complete medium was adjusted to pH 8.2 with NaOH. After resuspending the samples, cells were incubated at 25°C for the desired time during which gametogenesis took place. At the various time points, gametes could be observed alive or fixed for 30 min at room temperature in 1% paraformaldehyde. After either sedimentation for two hours at room temperature or centrifugation at 1,000 g for 1 min, stained cells were resuspended at 50% haematocrit in the same medium, mounted in a microscope slide under a sealed coverslip. In the analysis of live cells the sample was transferred to ice until observation to minimize metabolic activity in the parasites. Gametes were identified in bright field as round, pigmented objects with a diameter of about 5 μm and then evaluated for the presence or absence of WGA surface fluorescence, diagnostic respectively of the gamete intracellular or extracellular state. Percent of egress in the experiments described here was measured counting a minimum of 100 gametes per sample. A Leica DMRB microscope was used to visualize live samples. Images were acquired using a Leica DFC340 FX camera through a Leica PL FLUOTAR 40x objective. Filters used to detect Alexa 488 fluorescence were: EX: 515–560, EM: 590 long-pass filter. A Deltavision Elite microscope was used to visualize fixed samples. Images were acquired using a Coolspnap HQ2 CCD camera through an Olympus 100X UPlanSApo NA1.4 objective. Filters used to detect Texas Red fluorescence were: EX 575/25, EM 632/60. Images were processed using ImageJ 1.46r (NIH).

## Results and discussion

### Egress of *Plasmodium falciparum*gametes from fluorescently labelled erythrocytes

**Figure 1:**
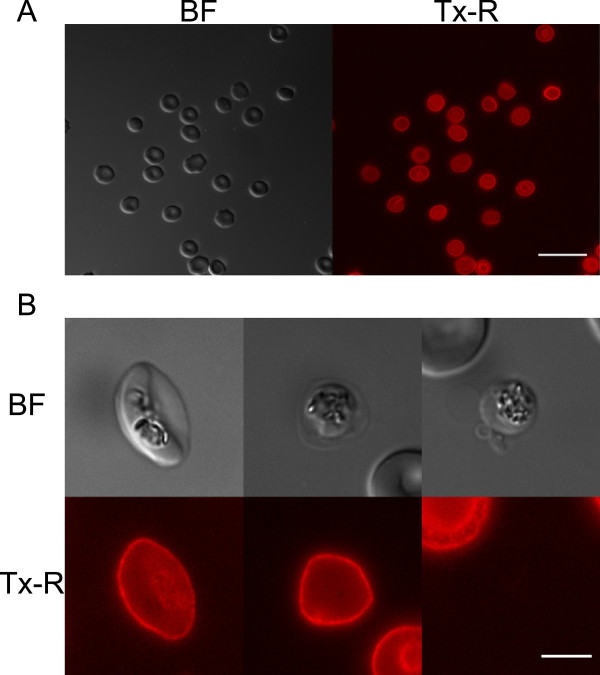
**Wheat germ agglutinin**-**stained uninfected and infected red blood cells. A)** Human uninfected RBCs stained by WGA-conjugated Texas Red observed in bright field and under UV light. Scale bar: 20 μm. **B)** Mature gametocyte (left panel), non-egressed gamete (centre), egressed gamete (right panel) stained with WGA-conjugated Texas Red. The Laveran’s bib can be seen in the gametocyte as a brighter fluorescent area. Scale bar: 5 μm. BF: bright field. Tx-R: Texas Red.

In order to restrict to a minimum the manipulation of the sexual stage parasites before and after gametogenesis, it was decided to obtain mature gametocytes contained in fluorescently stained erythrocytes. WGA covalently linked to fluorescent dyes such as Texas Red or Alexa 488 was chosen for this purpose. As it was previously reported that WGA staining increases adhesive properties and rigidity of RBCs [13], it was preliminarily established that using WGA concentration up to 5 μg/ml with volumes between 0.1 to 1 ml of blood at 3-4% haematocrit did not produce cell clumping. A 15-min incubation of uninfected and infected erythrocytes at 37°C with 5 μg/ml fluorescent WGA was sufficient to obtain cell samples in which virtually all erythrocytes showed a clear and homogeneous fluorescent signal on their surface (Figure 1A). The signal showed a virtually indistinguishable intensity on the surface of uninfected and infected RBCs, also in the case of erythrocytes deformed by the elongated mature gametocytes (Figure 1B, left panel).

**Figure 2:**
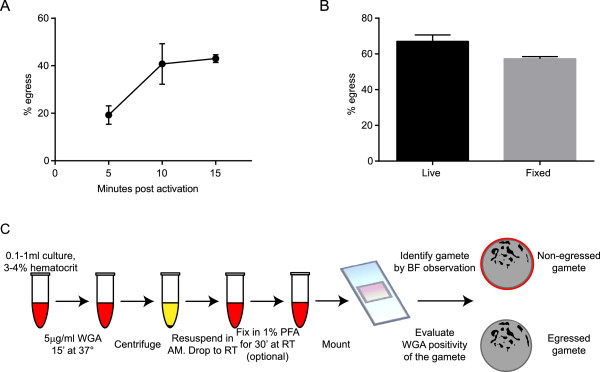
**Validation and flow chart of the wheat germ agglutinin**-**staining protocol. A)** Time course of gamete egress at 5, 10 and 15 min after the addition of 20 μM XA. Error bar is SD from four biological replicates. **B)** Effect of cell fixation in the measurement of gamete egress. Data from three independent experiments, error bar is SD. **C)** Flow chart of the WGA-staining protocol. WGA: wheat germ agglutinin, AM: activation medium, RT: room temperature, PFA: paraformaldehyde, BF: bright field.

In order to measure egress, synchronous stage V gametocyte cultures (routinely 2-3% gametocytaemia) were stained as described above. A 0.5 ml sample of the stained gametocyte culture was centrifuged for 1 min and the pelleted RBCs were immediately resuspended at 25°C in complete medium with 20 μM XA to trigger rounding up and gamete formation. Parasites were analysed between 5 and 15 min post activation, and spherical gametes were readily identified at all time points in bright field microscopy, as expected. Inspection of the spherical parasites with appropriate filters enabled to clearly distinguish cells still surrounded by the fluorescent WGA signal, which were scored as intracellular gametes (Figure 1B, central panel), from those devoid of any fluorescence, suggesting that they had shed the surrounding erythrocyte membrane (Figure 1B, right panel). A time course measuring the two classes of parasites indicated that gamete egress reaches a plateau within 10–15 min from induction (Figure 2A), showing comparable kinetics and efficiency as measured in previously published experiments [5, 7, 12]. Finally, in order to rule out that free WGA could artifactually decorate the surface of extracellular gametes, a gametocyte culture was divided in two aliquots, one stained with WGA and one unstained. Both were induced to gametogenesis and the latter sample was stained with WGA after 15 min. Counts from pre-stained and post-stained triplicate samples were very similar, respectively measuring a 61.3% (SD: 6,6) and a 60,3% (SD: 3,3) in egress efficiency. In both cases only uninfected RBCs and the residual intracellular gametes were surrounded by the fluorescent signal.

These experiments indicate that mature gametocytes inside fluorescent WGA-stained erythrocytes are able to undergo rounding up and to egress with the expected kinetics and efficiency, and that gametes can be sampled and inspected without further delay after induction to assess their intracellular or extracellular status.

### Cell fixation does not interfere with gamete egress and ensures cell sample storage

The above experiments provide the basis for an easy, fast and minimally invasive protocol to visualize and measure gamete egress. This protocol is suitable for live imaging applications, time course experiments and determination of dose response effects of treatments or compounds on this process. In order to improve the throughput of the protocol with the possibility to store parasite samples for subsequent analysis, measurements were compared of gamete egress in parallel samples of gametes, sampled at 15 min from induction, treated or untreated with 1% paraformaldehyde for 30 min. Results showed that number of egressed gametes counted in the fixed parasite samples was slightly reduced compared to that measured in the unfixed cells, possibly suggesting that a small fraction of unfixed gametes manages to egress during the sample storage time (Figure 2B). These experiments therefore showed that a cell fixation step provides the important possibility to store samples for subsequent analysis and was for this reason introduced in the flow chart of the protocol (Figure 2C).

To evaluate performance of the newly established methodology, this protocol was applied in a series of experiments addressing distinct aspects of gamete egress.

### Live imaging of erythrocyte disruption at gamete egress

**Figure 3:**
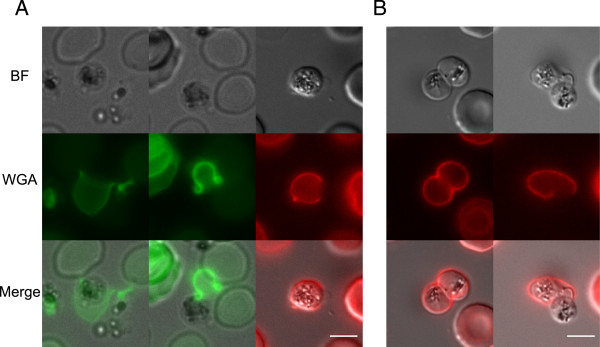
**Red blood cell membrane disruption in gametes egress. A)** Gamete egress observed on live (left and central panels, WGA-Alexa488 staining) and fixed (right panel, WGA-Texas Red staining) parasites. **B)** Two gametes within the same red blood cell. The host cell shows in one case an intact membrane (left panel) and a single opening at the start of gamete egress (right panel). Scale bars: 5 μm. BF: bright field. WGA: wheat germ agglutinin.

The newly established protocol provides the opportunity to follow the events of gamete egress in live parasites. A few live microscopy descriptions exist of this process, although lack of specific markers constrained the unambiguous identification of the membranes involved. For instance, live observations in *Plasmodium gallinaceum* proposed that in female gamete egress, unlike in male exflagellation, the RBC membrane ruptures before the PVM [14], an observation which was not confirmed by subsequent ultrastructural work on female gametes, which supported instead an ‘inside-out’ egress mechanism [6, 15–20]. As in some of these studies extracellular gametes were observed by ultrastructure [6, 19] or optical microscopy [20] next to erythrocyte ghosts showing a single large opening, live observations were performed using the new protocol to investigate this point in more detail. In these experiments it was consistently observed that spherical gametes indeed gain access to the extracellular milieu through a single exit point in the infected erythrocyte membrane (Figure 3A), confirming this egress mechanism [6]. Amongst the at least 50 events observed, it was remarkable to see that also in the few cases of erythrocytes co-infected by two gametocytes, the egress of both spherical gametes was clearly occurring from a single opening induced in the RBC membrane (Figure 3B). These results highlight an intriguing similarity between the egress of gametes and that of merozoites, despite the different size of the two parasite stages. It is described that the burst of the asexual schizont is triggered by the egress of an individual merozoite through a single disruption point induced in the erythrocyte membrane [21]. In the gamete egress experiments erythrocyte ghosts could be occasionally detected whose morphology is intriguingly reminiscent of that of erythrocyte membranes ‘curling’ upon merozoite release (Figure 3A) [21], leading to speculate that mechanistic aspects of egress may be shared by asexual and sexual stages.

The new protocol enables live observations also of the comparatively less frequent event of male gamete egress. In preliminary observations on 30 male gamete activation events, in 25 cases the exflagellation centres were not associated with a WGA fluorescent signal, whereas in the remaining five cases some of the newly formed gametes were still transiently associated to the erythrocyte ghosts.

As a further improvement, the newly established protocol could be used in live imaging application with gametocytes in which the PVM is fluorescently labelled by specific reporter proteins [20]. This may provide the possibility to obtain a dynamic description of the disruption of both membrane compartments surrounding the rounded up gamete. So far, a clear distinction between disruption of the two membranes has been chiefly achieved by examination of ultrastructural sections [7, 9].

### Egress of osmiophilic body-depleted female gametocytes

Given its suitability to reliably measure female gamete egress, the protocol was used to examine the only *P. falciparum* mutant whose gametogenesis was reported to be affected only in this sex. Pfg377 is the only protein described to specifically reside in electron dense organelles named osmiophilic bodies (OBs), which preferentially accumulate in female gametocytes [22, 23]. Disruption of the *pfg377* gene results in female gametocytes with a dramatically reduced content of these organelles. Experiments analysing egress of the OB-depleted female gametocytes showed a two-fold reduction in egress efficiency at 15 min from induction in the mutant parasites compared to the parental line [5]. This suggested a possible, albeit non-essential, role of such organelles in this process, also supported by the observation that OBs are no longer detectable as soon as female gametes are formed.

**Figure 4:**
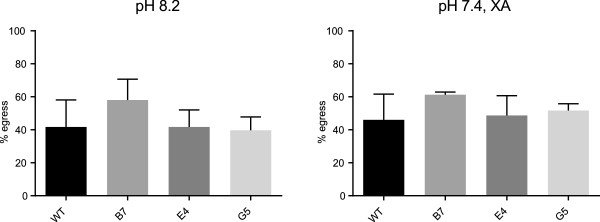
**Egress efficiency in** ***wt*** **and** ***pfg377KO*** **gametes.** Egress efficiency of gametes from *wt* and three *pfg377KO* clones after induction with pH 8.2 and pH 7.4 plus 20 μM XA, error bar is SD.

In order to further analyse this point, the egress efficiency of female gametes was re-assessed with the new protocol in the three previously published *pfg377KO* clones, from two independent gene disruption experiments, and in their parental parasites [5]. Egress was measured at 15 min post induction in nine independent experiments, in six cases triggered by raise in pH only and in three by XA only. In all cases, the result was that the previously described two-fold reduction in egress efficiency could not be reproduced (Figure 4). This discrepancy could be explained as follows: in the previous report extracellular gametes were positively identified as fixed, non-permeabilized spherical parasites whose surface was stained by a monoclonal antibody against the gametocyte/gamete surface antigen Pfs230. As in fact was considered in that publication, it is conceivable that the depletion of OBs and/or absence of Pfg377 does not reduce efficiency of egress from the erythrocyte of the mutant gametes but rather it impairs the reactivity of their surface to the anti-Pfs230 antibody, leading to an underestimate of egress efficiency. Examination of *pfg377*KO gamete egress with the new protocol does not support the hypothesis of a major role of OBs in the ability of female gamete to disrupt and shed the erythrocyte membrane during egress. As however the Pfg377-defective parasites showed a dramatic defect in vector transmissibility [5], the above results rather propose that OB discharge and/or the associated Pfg377 mobilization play a functional role in subsequent steps leading to gamete fertilization or in further parasite development in the mosquito.

## Conclusions

Studying the egress of *P. falciparum* gametes is important to understand the mechanisms of transmission of the malaria parasite to its mosquito vector. The protocol presented here provides a quantitative method for the fast and simple determination of egress of *Plasmodium* gametes from the infected RBCs. This method is applicable to *in vivo* imaging and, as gametes can be fixed and stored, to higher throughput analyses, such as the screening of compounds inhibiting this key process of the parasite life cycle.

## Abbreviations

WGA: Wheat germ agglutinin
OBs: osmiophilic bodies
XA: xanthurenic acid
RBC: red blood cell
PVM: parasitophorous vacuole membrane.
